# The Role of Medium Polarity in the Efficiency of Albumin Binding with Hydrophobic Ligands: Experimental Studies and a Molecular Dynamics Investigation

**DOI:** 10.3390/ijms252312664

**Published:** 2024-11-25

**Authors:** Gabriel Zazeri, Ana Paula Ribeiro Povinelli, Luiza de Carvalho Bertozo, Alan M. Jones, Valdecir Farias Ximenes

**Affiliations:** 1 Departamento de Física, Universidade Federal de Roraima (UFRR), Boa Vista 69310-000, RR, Brazil; gabriel.zazeri@ufrr.br; 2 Instituto de Biociências Letras e Ciências Exatas, Universidade Estadual Paulista (UNESP), São José do Rio Preto 15054-000, SP, Brazil; ana.povinelli@unesp.br; 3 Faculdade de Ciências, Universidade Estadual Paulista (UNESP), Bauru 17033-360, SP, Brazil; luiza_bertozo@yahoo.com.br; 4 School of Pharmacy, Institute of Clinical Sciences, College of Medical and Dental Sciences, University of Birmingham, Birmingham B15 2TT, UK

**Keywords:** hydrophobic effect, entropy, albumin, 1,6-diphenyl-1,3,5-hexatriene, octyl gallate, quercetin, rutin

## Abstract

This study evaluates how the polarity of the medium affects the binding efficiency of hydrophobic ligands with human serum albumin (HSA). The polarity of the aqueous medium was changed by adding 1,4-dioxane in concentrations of 0%, 10%, and 20% *w*/*w*, resulting in solvent mixtures with decreasing dielectric constants (ε = 80, 72, and 63). The addition of 1,4-dioxane did not affect the integrity of the protein, as confirmed by Far-UV-CD, Rayleigh scattering, and time-resolved fluorescence experiments. The impact of medium polarity on the binding constants was evaluated using 1,6-diphenyl-1,3,5-hexatriene (DPH), octyl gallate (OG), quercetin, and rutin as ligands. The association constants of DPH decreased as the medium hydrophobicity increased: at 0%, *Ka* = 19.8 × 10^5^ M^−1^; at 10%, *Ka* = 5.3 × 10^5^ M^−1^; and at 20%, *Ka* = 1.7 × 10^5^ M^−1^. The decrease was still higher using OG: at 0%, *Ka* = 5.2 × 10^6^ M^−1^; and at 20%, *Ka* = 2.2 × 10^5^ M^−1^. The results in the same direction were obtained using quercetin and rutin as ligands. Molecular dynamics simulations illustrated the hydrophobic effect at the molecular level. The energy barrier for DPH to detach from the protein’s hydrophobic site and to move into the bulk solution was higher at 0% (9 kcal/mol) than at 20% 1,4-dioxane (7 kcal/mol). The difference was higher for OG, with 14 and 6 kcal/mol, respectively. Based on these findings, it was shown that the difference in hydrophobicity between the protein’s microenvironment and the surrounding solvent is an essential component for the effectiveness of the interaction. These results shed light on albumin–ligand complexation, a molecular interaction that has been extensively studied.

## 1. Introduction

Fat is the primary fuel source when engaging in physical activity lasting longer than two hours. The muscles utilize non-esterified fatty acids from the bloodstream, which are transported by human serum albumin (HSA), to provide energy during exercise [1]. This fact highlights the crucial role of HSA, a transporter for poorly soluble molecules in the bloodstream. This includes the transport of fatty acids from adipose tissue to the muscles, among other essential physiological processes [2,3]. Equally important is its role as a drug carrier, which directly affects pharmacokinetics and pharmacodynamics [3,4,5,6,7]. An advancement in this field involves developing drugs bound to HSA for intraperitoneal drug delivery, known as albumin-based cancer therapeutics [8]. An example of this is abraxane, an exogenous HSA-bound paclitaxel formulation approved by the FDA and used to treat locally advanced or metastatic tumors [9].

The preceding paragraph emphasizes one of the biological characteristics of HSA, rooted in the physics of the hydrophobic effect, which is fundamental in entropy-driven interactions [10]. Given the importance of thermodynamic properties in this study, let us review some key concepts and how they relate to the binding of albumin and drugs. The increase in entropy determines the spontaneity of physical, chemical, and biological processes. However, in an isolated system, a decrease in entropy can occur if it is offset by an enthalpic factor, resulting in an overall reduction in Gibbs free energy. In the context of biomolecular interactions, this phenomenon is often associated with enthalpy–entropy compensation [11,12,13]. Numerous research papers have reported interactions between albumin and ligands where the enthalpy (ΔH < 0) drives a spontaneous reaction (ΔG < 0) despite a decrease in overall entropy (ΔS < 0). In these cases, it is generally argued that hydrogen bonds and van der Waals forces between ligands and the amino acid residues in the binding sites play a crucial role in stabilization [14,15,16,17]. Conversely, entropic gain also dictates many complexations [18,19,20]. The Gibbs free energy equation correlates these thermodynamic parameters and clarifies why ΔH < 0 and ΔS > 0 contribute to a favorable binding ΔG < 0 (Equation (1)).

(1) $$∆{G{}^{\circ}}^{\prime }=∆{H{}^{\circ}}^{\prime }-T∆{S{}^{\circ}}^{\prime }$$

The overall change in entropy due to protein–ligand complexation is the summation of several components. Contributing negatively, one component has entropy loss due to decreased rotational and translational degrees of freedom in the complex relative to the unbound species. This is typical of complexation reactions, where two or more reactant molecules come together to form the product [21,22]. Contributing positively to the binding constant, one has entropic gain usually associated with the hydrophobic effect, also called the entropy change in the solvent or solvation entropy [23,24,25]. Regarding protein–ligand binding, this relates to the ligand displacement from the aqueous bulk solvent to the hydrophobic cavities in the protein. In addition, it must include the release of water from the active site upon ligand binding [23]. Finally, one has the conformational entropy change due to the changes in the conformational freedom of both the protein and ligand upon binding, which may contribute favorably or unfavorably [21].

In the context mentioned above, the entropic factor is crucial in determining the thermodynamic feasibility of albumin–ligand binding. Typically, the change in solvent entropy accounts for a significant portion of the overall entropy [24]. Given this fact, we sought to investigate how manipulating the solvent polarity would impact the effectiveness of HSA as a drug carrier. Instead of modifying the molecular structure of the ligand and observing its effect on the binding constant, we opted to alter the solvent polarity. The interactions of hydrophobic ligands such as 1,6-diphenyl-1,3,5-hexatriene and octyl gallate with HSA were evaluated for this task. Our findings indicate that the binding efficiency of HSA is reliant on the polarity of the medium.

## 2. Results and Discussion

### 2.1. Integrity of HSA in the Presence of 1,4-Dioxane

**Figure 1:**
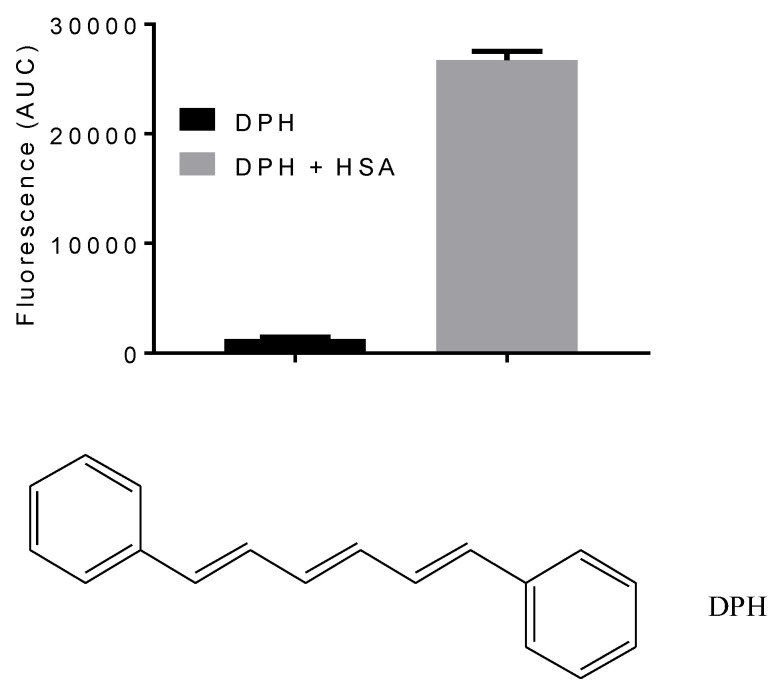
The molecular structure of 1,6-diphenyl-1,3,5-hexatriene (DPH) and the increase in fluorescence due to adding HSA. Excitation at 370 nm and emission in the 400 to 600 nm range. DPH 1.0 μM, HSA 10 μM. The results are expressed as mean and SD of triplicates.

DPH was selected to assess the impact of medium polarity on ligand binding efficiency with HSA. DPH is a commonly used fluorescent probe in lipid membrane studies, micellar systems, and macromolecules [26,27,28,29,30]. It has low aqueous solubility, and its aggregation in these mediums precludes its detection by fluorescence, which is a typical feature of the water-insoluble fluorescent compound [31]. However, when DPH is transferred to a hydrophobic environment, it emits light with high quantum efficiency. This behavior was observed when HSA was added to the previously DPH-containing aqueous medium (Figure 1). Based on this feature of the DPH and HSA interaction, we developed the following experimental approaches to evaluate the role of medium polarity:

- To determine the binding constant of DPH in solvents of different polarities (mixtures of water and 1,4-dioxane);
- To determine the effect of NaCl;
- To evaluate the transfer of DPH from the protein environment to the bulk phase by molecular dynamics.

In short, the proposal was to alter the medium polarity while keeping the protein and ligands constant; hence, changes in the binding efficiency could be correlated with alterations in the hydrophobic effect related to the transfer of the ligand from the bulk phase to the protein microenvironment.

**Figure 2:**
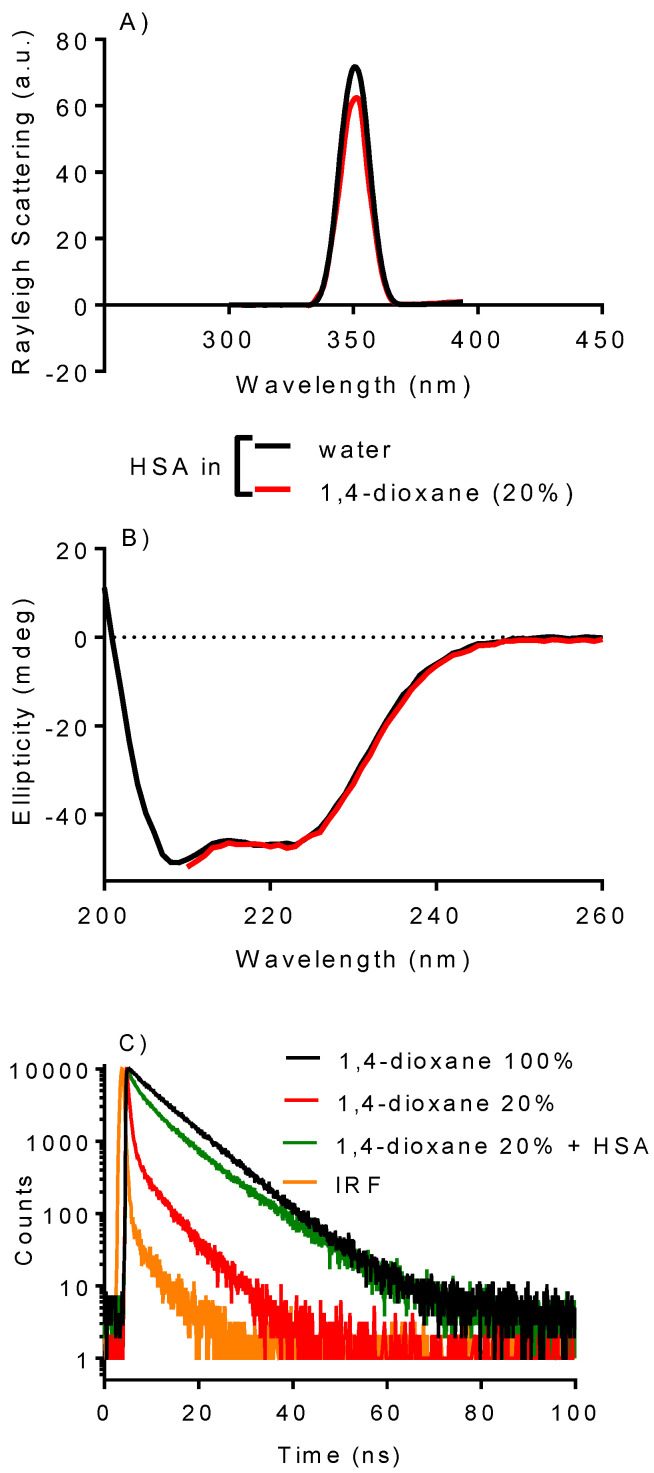
Effect of 1,4-dioxane (20%) on HSA structure examined by (**A**) aggregation (Rayleigh peak) and (**B**) secondary structure (far-UV-CD). Experimental condition HSA (2.0 μM) in pure water or 1,4-dioxane (20%). (**C**) Time-dependent fluorescence decay of DPH in 1,4-dioxane and complexed with HSA. Experimental condition: DPH 0.2 μM, HSA 10.0 μM in water with or without 1,4-dioxane (20%).

The decision to use 1,4-dioxane was based on two key factors. Firstly, the dielectric constant, which measures the medium’s polarity, has been documented for mixtures of 1,4-dioxane and water [26,32,33]. Secondly, there is evidence that this solvent has minimal impact on albumin at concentrations up to 35% in water [34]. Notwithstanding, and even using 20% of 1,4-dioxane at most before measuring the binding constants, we assessed the protein’s integrity under the experimental conditions. Figure 2A shows the Rayleigh scattering spectra of HSA in water and with 1,4-dioxane (20%). The protein misfolding can be studied using this assay since increased protein aggregation causes light scattering at 350 nm [35,36]. As shown, the peak was slightly changed, meaning the solvent did not cause significant protein clumping. Additionally, the Far-UV-CD spectrum of HSA was not affected by the solvent (Figure 2B), meaning that the basic secondary structure of HSA remained the same. It is important to note that we could only take measurements above 210 nm because the solvent strongly absorbed light, making it impossible to record the spectrum below this value.

Further evidence supporting that the presence of 1,4-dioxane did not hinder the protein from functioning as a drug carrier was acquired by measuring the fluorescence lifetime of DPH. It was shown that DPH has an average lifetime of 7.8 nanoseconds in pure 1,4-dioxane, which is consistent with the typical values found in solvents (Figure 2C) [37]. In 20% 1,4-dioxane, the average value decreased to 2.6 nanoseconds, and a noticeable multi-exponential decay pattern was observed. This is the typical behavior of low-solubility fluorescent probes in aqueous mediums [38,39]. However, when the protein was added to the medium, the lifetime increased to 7.1 nanoseconds, implying that DPH was experiencing a microenvironment similar to pure 1,4-dioxane (Figure 2C). In summary, these findings suggest that HSA can form a complex with DPH even in the presence of 20% 1,4-dioxane, indicating that the protein’s capacity for complexation remained functional.

### 2.2. Effect of 1,4-Dioxane on the Association Constant of DPH

**Figure 3:**
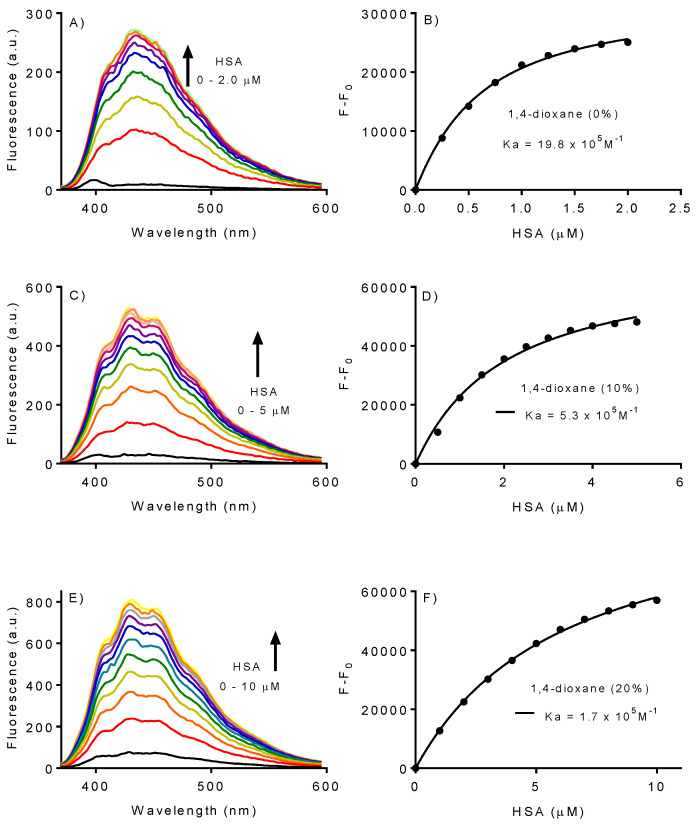
Effect of 1,4-dioxane on the association constants between DPH and HSA. (**A**,**B**) Titration of DPH with HSA in water. (**C**,**D**) Titration of DPH with HSA in 1,4-dioxane (10%). (**E**,**F**) Titration of DPH with HSA in 1,4-dioxane (20%). The association constants were obtained by fitting the data in Equation (2).

Once HSA was not significantly affected by the presence of 1,4-dioxane, its ability to bind with DPH was tested. The experiments involved titrating a fixed concentration of DPH with HSA. The binding constants were determined in solutions containing 0%, 10%, and 20% of the solvent. The increase in fluorescence resulting from the formation of complexes was used as an analytical parameter, and the binding constants were calculated by fitting the data to Equation (2). The graphs in Figure 3 illustrate the typical behavior of complex formation, where the fluorescence reaches a maximum at the protein’s saturation concentration. However, the experiment clearly showed a significant difference regarding the percentage of 1,4-dioxane. The fluorescence increased and the saturation occurred at a 5-fold lower concentration of HSA when the solvent was absent. For example, while 10 μM of HSA was needed to reach saturation using 1,4-dioxane (20%), only 2.0 μM was required in pure water (Figure 3A,C). The consequence was a decrease in the binding constant in the presence of the solvent (Figure 3B,D,F).

**Figure 4:**
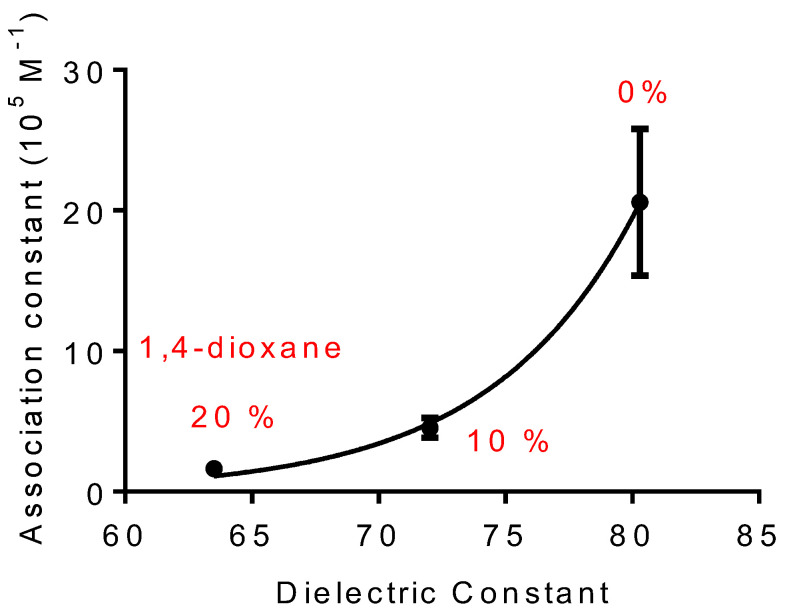
The relevance of bulk solvent dielectric constant on binding constants between DPH and HSA. The dielectric constant of water was modified by adding 1,4-dioxane. The results are the means and SD of triplicates. *Ka* (20%) 20.6 ± 5.2 × 10^5^ M^−1^, (10%) 4.5 ± 0.7 × 10^5^ M^−1^, and (0%) 1.7 ± 0.3 × 10^5^ M^−1^.

Figure 4 summarizes the findings and illustrates the relationship between the medium’s polarity and the association constants. As can be seen, the association constant was inversely related to the percentage of 1,4-dioxane, emphasizing the medium’s importance for binding effectiveness.

### 2.3. Effect of NaCl on the Association Constant of DPH

**Figure 5:**
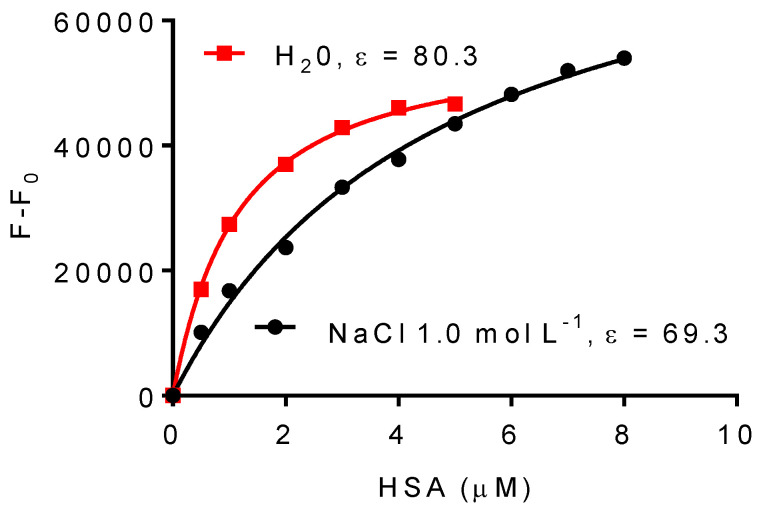
The effect of NaCl solution on the association constants between DPH and HSA. The dielectric constant of water was modified by adding NaCl 1.0 mol L^−1^. The association constants were obtained by fitting the data in Equation (2). *Ka* (NaCl 1.0 mol L^−1^) 2.3 ± 0.3 × 10^5^ M^−1^, (H_2_O) 9.9 ± 0.5 × 10^5^ M^−1^.

As stated above, using 1,4-dioxane in protein solution was justified based on the literature data [34] and confirmed by our experiments (Figure 3). However, to reinforce and unlink our findings from a specific property of 1,4-dioxane, the alteration in the bulk solvent dielectric constant was also accomplished by another method, i.e., by adding NaCl. This was possible due to the relationship between NaCl concentration and the dielectric constant of the aqueous solution. Specifically, the binding constant of DPH was determined using 1.0 mol/L NaCl, which has a dielectric constant of 69.3 [40,41] as opposed to when it is in pure water (80.3). The result in Figure 5 confirmed the previous one, i.e., the binding constant of DPH was lower in a lower polarity medium. The binding constant of DPH in NaCl solution was (2.3 × 10^5^ M^−1^) and in pure water (9.9 × 10^5^ M^−1^).

### 2.4. Effect of 1,4-Dioxane on the Association Constant of Octyl Gallate

**Figure 6:**
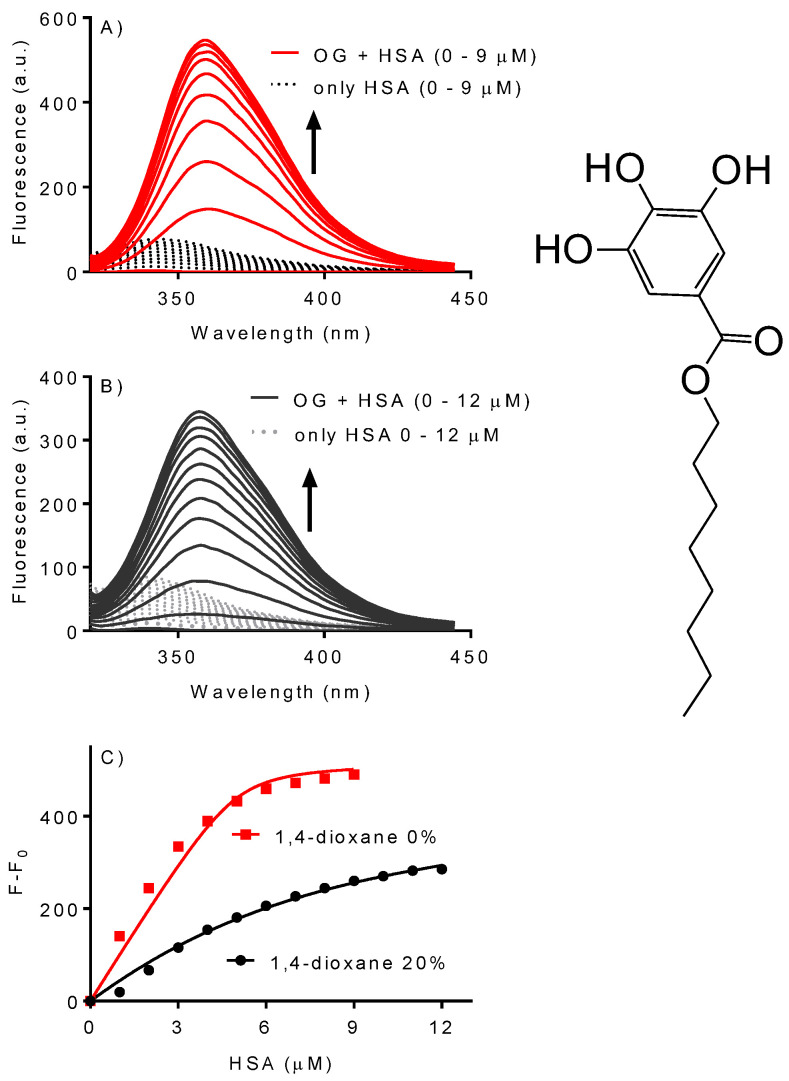
Effect of 1,4-dioxane on the association constants between octyl gallate (OG) and HSA. (**A**) Titration of OG with HSA in water. (**B**) Titration of DPH with HSA in 1,4-dioxane (20%). (**C**) The association constants were obtained by fitting the data in Equation (2). *Ka* (20%) 2.2 ± 0.6 × 10^5^ M^−1^, and (0%) 5.2 ± 2.5 × 10^6^ M^−1^.

Additional ligands were further tested to explore how medium polarity affects albumin binding. The impact of the food additive octyl gallate (OG) was studied explicitly for this purpose. Because OG has a hydrophobic eight-carbon chain (Figure 6), the medium’s polarity was expected to affect it. Additionally, OG’s fluorescence increases upon binding with HSA [42], allowing for the measurement of the binding constant, as illustrated in Figure 6. The presence of 1,4-dioxane significantly impacted the binding constant, surpassing the effects observed for DPH and resulting in a 25-fold increase in pure water compared to complexation in the presence of the organic solvent. Specifically, the binding constant of OG in 20% 1,4-dioxane was 2.16 × 10^5^ M^−1^, whereas in pure water, it was 5.15 × 10^6^ M^−1^.

### 2.5. Quercetin Versus Rutin as Ligands of HSA

**Figure 7:**
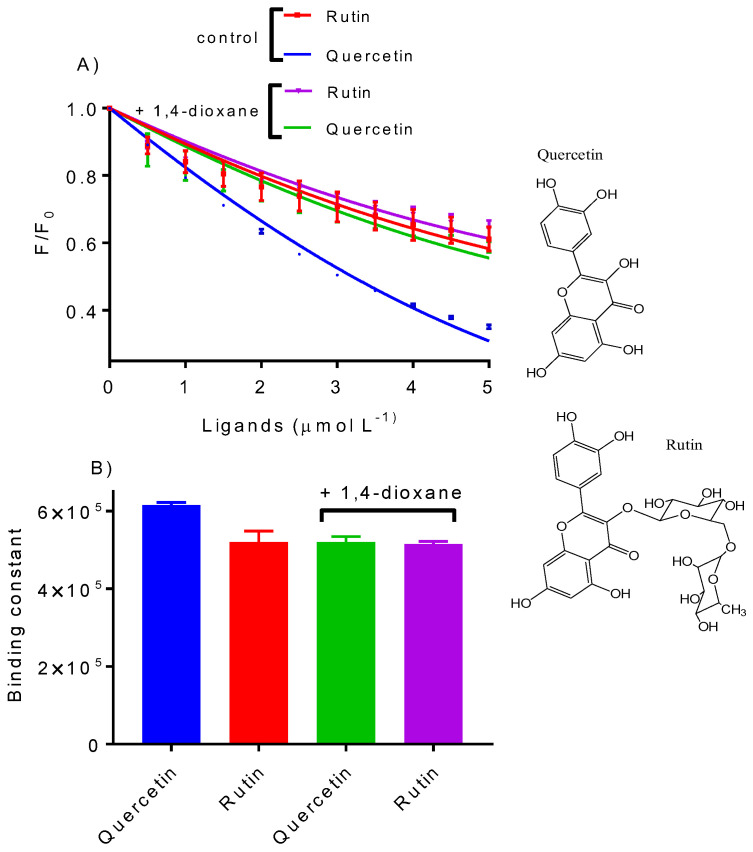
The sensitivity of quercetin and rutin to 1,4-dioxane. The association constants were determined in the absence (control) and presence of 1,4-dioxane (20%). (**A**) Isotherms for the determination of binding constants were fitted using Equation (3). (**B**) Binding constants in the presence and absence of 1,4-dioxane.

A different experimental approach was developed to highlight the significance of medium polarity on binding efficiency. This involved specifically modifying the ligand hydrophobicity while maintaining the medium. For this task, the binding strength of quercetin and rutin were compared. Quercetin and rutin are commonly used flavonoids, and their binding with HSA has previously been studied [43,44]. Both contain a 3-hydroxyflavone group but differ in the presence of the disaccharide rutinose (rutin) or its absence (quercetin) (Figure 7). Rutin, with the sugar group, is more water-soluble (125 mg/L) and hydrophilic (logP = −2.28) compared to quercetin (60 mg/L, logP = 0.35). Based on our hypothesis, we expected the medium effect on the binding strength to be more significant for quercetin than rutin. In Figure 7, we show the measurement of the binding strength in pure water and in a 20% 1,4-dioxane solution. Unlike the previous experiments, the binding strengths were determined by the effect of the ligands on the protein’s natural fluorescence. As depicted, 1,4-dioxane decreased quercetin’s binding strength but not rutin. This result confirms our hypothesis by demonstrating the higher sensitivity of the more hydrophobic molecule to medium polarity.

### 2.6. Molecular Dynamics Studies

**Figure 8:**
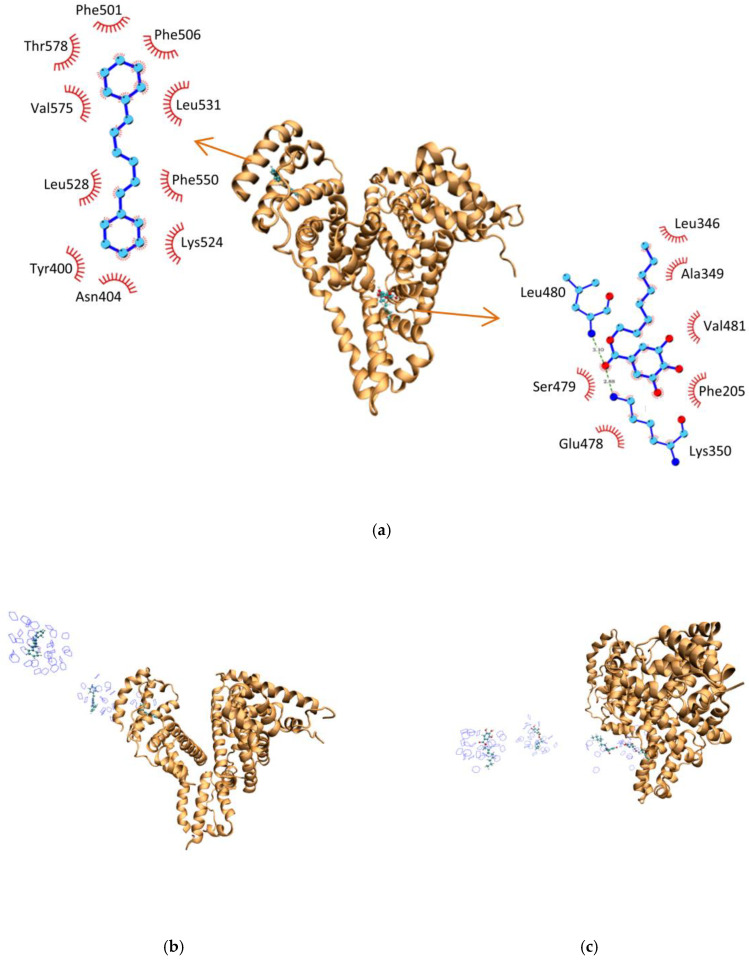
Structure of molecules bound to albumin: (**a**) The left diagram illustrates the amino acids responsible for hydrophobic interactions with DPH when bound to HSA. The right diagram displays the amino acids involved in hydrophobic interactions and hydrogen bonding (Leu480 and Lys350) with OG when bound to HSA. (**b**) The pulling of DPH away from HSA in the simulation with 20% of dioxane (shown in blue around DPH). (**c**) The pulling of OG away from HSA in the simulation with 20% of dioxane (shown in blue around OG).

The DPH molecule is stabilized in the binding site of HSA (Figure 8a) through the hydrophobic interactions with Phe (501 and 550), Leu (528 and 531), Val575, and Tyr400. The OG molecule is stabilized in the binding site of HSA by the hydrophobic interactions with Leu346, Ala349, Val481, and Phe205, as well as by hydrogen bonds with the nitrogen of Leu480’s main chain and the nitrogen of Lys350’s side chain.

**Figure 9:**
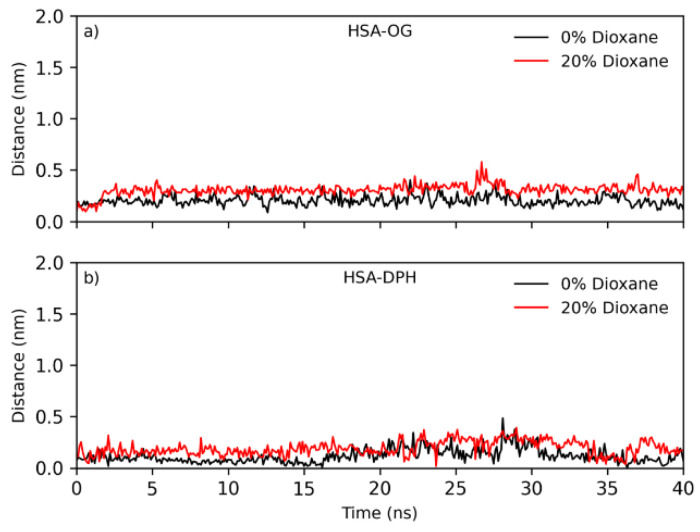
Distance between the center of mass of the protein binding site and the center of mass of (**a**) octyl gallate and (**b**) DPH in 0% (in black) and 20% (in red) of 1,4-dioxane in the solvent.

The stability of the albumin–ligand complex was analyzed during 40 ns of simulation; Figure 9 shows the distance from the center of mass of the ligands to the center of mass of the albumin binding site throughout the simulation. According to the results, both DPH and OG remained stable within the binding site (distance remained around 0.2 nm), with slight fluctuations in 0% and 20% of 1,4-dioxane.

**Figure 10:**
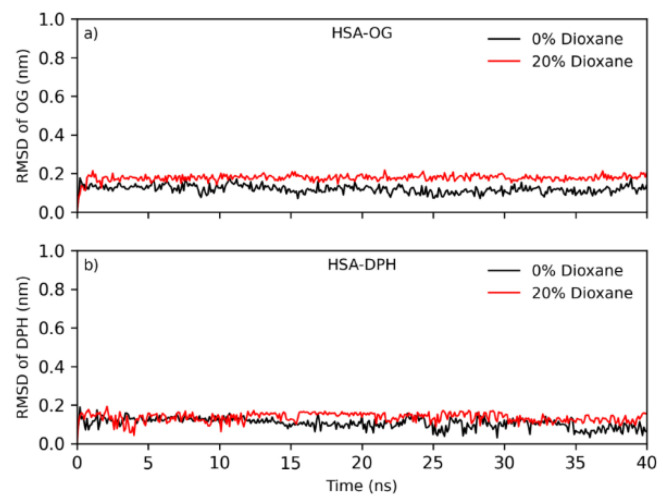
RMSD of the ligands (**a**) OG and (**b**) DPH in the protein binding site at concentrations of 0% (in black) and 20% (in red) of 1,4-dioxane.

Figure 10 shows the RMSD graph of the center of mass of the ligands in the protein binding site during 40 ns of simulation in both 0% and 20% of 1,4-dioxane. According to the results, ligand stability within the site is observed again, where the RMSD (around 0.2 nm) of both ligands remained stable with minor fluctuations. These results indicate that the ligands remain stable in the binding sites when the systems were simulated with 0% and 20% of dioxane.

To verify the difference in binding affinity caused by the hydrophobic effect at a molecular level, molecular dynamics simulations using the umbrella sampling method were performed. The molecules of DPH and OG were pulled from their binding site in systems composed of 0% and 20% of dioxane (shown in Figure 8b,c).

**Figure 11:**
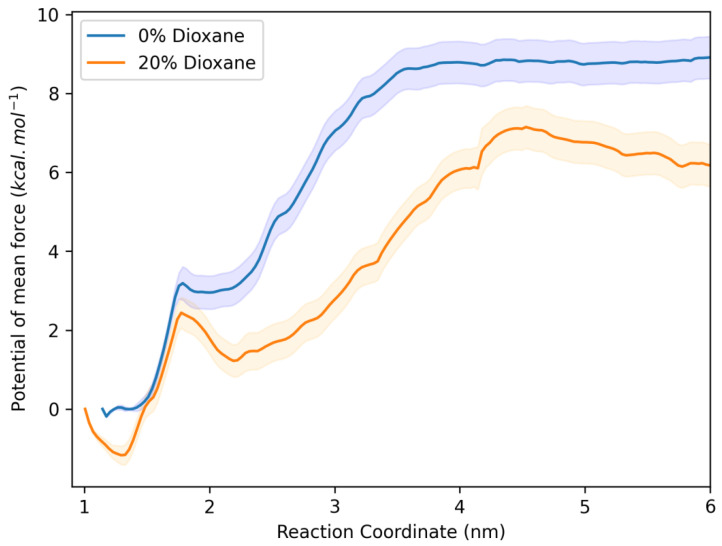
Potential of mean force (PMF) for the dissociation of DPH from HSA at both solvents: 0% (in blue) and 20% (in orange) of 1,4-dioxane.

The potentials of the mean forces were obtained using the WHAM method. Figure 11 shows the potential of the mean force plot of the DPH molecule and the HSA protein under conditions where the solvent contains 0% and 20% of 1,4-dioxane. According to the results, it can be observed that the energy barrier for the DPH molecule to lose interaction contact with the hydrophobic site of the protein and move into the bulk solvent is higher (~9 kcal/mol) when the medium is less hydrophobic (0%) than when it is more hydrophobic (~7 kcal/mol in 20%). After conversion, this corresponds to an 8.4 kJ/mol difference. This result highlights that when the solvent is less hydrophobic, the DPH has a lower energy cost to be directed to the protein’s interaction site, which is more hydrophobic than the solvent, thus demonstrating the hydrophobic effect.

**Figure 12:**
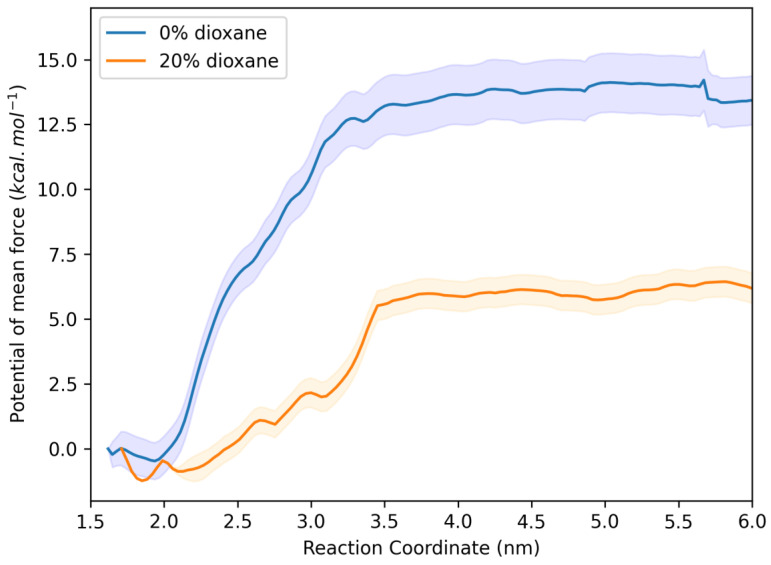
Potential of mean force (PMF) for the dissociation of OG from HSA at both solvents: 0% (in blue) and 20% (in orange) of 1,4-dioxane.

In addition to analyzing the DPH molecule, we evaluated this effect with OG. Figure 12 shows plots with similar profiles to those in Figure 11, but the hydrophobic effect is even more pronounced in this case. Here, the energy barrier for the OG molecule to lose contact with interaction from the protein site is higher (~14 kcal/mol) in the medium without 1,4-dioxane than in the medium with 20% 1,4-dioxane (~6 kcal/mol). In this case, the energy difference between the two barriers is 33.44 kJ/mol, further highlighting the hydrophobic effect.

In both cases, we observed that in systems where the solvent is more hydrophobic, the DPH and OG molecules have a lower preference for the protein’s hydrophobic site, as the energy barrier to detach these ligands from the protein is much lower than in cases where the solvent is less hydrophobic. This result and the experimental findings demonstrate that the hydrophobic effect is essential in directing the protein–ligand interaction forces.

The studies by Sarter et al. [45], Verteramo et al. [46], and Syme et al. [47] emphasize that, in some systems, solvent dynamics and conformational flexibility can meaningfully affect binding thermodynamics through entropic contributions. In our case, however, molecular dynamics (MD) simulations revealed minimal changes in the root mean square deviation (RMSD) of the geometric center of the binding site and ligands across different solvent conditions, indicating that the additives did not significantly alter the structural stability or flexibility of the binding partners. Therefore, while such entropic contributions may be significant in other systems, our MD results suggest that they play a negligible role in the observed binding affinities in this study.

## 3. Materials and Methods

### 3.1. Reagents

Human serum albumin (HSA) free of fatty acid and globulins, 1,6-diphenyl-1,3,5-hexatriene (DPH), octyl gallate (OG), quercetin, rutin, sodium chloride, and 1,4-dioxane were purchased from Sigma-Aldrich Chemical Co. (St. Louis, MO, USA). Stock solutions of DPH, OG, quercetin, and rutin (10 mmol L^−1^) were prepared in DMSO. HSA was dissolved in PBS at pH 7.4 to give a 1.0 mmol L^−1^ stock solution, and its concentration was measured by UV-Vis absorption (ε_280nm_ = 35,219 mol^−1^ L cm^−1^) using Perkin Elmer Lambda 35 UV-visible spectrophotometer (Shelton, CT, USA). All solutions were prepared with water purified by a Milli-Q system (Millipore, Bedford, MA, USA).

### 3.2. Measurement of Binding Constants

The interactions of the DPH and OG with HSA were evaluated by the increase in the intrinsic fluorescence of the probes. DPH: the excitation was set at 350 nm, and the emission was 380 to 600 nm. The slit widths were set at 10 nm for both excitation and emission wavelengths, and a 3 mL quartz cuvette with a 10 mm path length and that was magnetically stirred was used during the measurements. The experiments were performed in pure water or solutions containing 1.0 mol L^−1^ NaCl, 10 and 20% (*w/w*) of 1,4-dioxane. The experiments were performed by titration of a fixed concentration of the DPH (0.2 µmol L^−1^) with HSA (0–10.0 µmol L^−1^) at 25 °C. After each addition, the mixtures were incubated for 2 min before the fluorescence measurements. Equation (2) was applied to determine the binding constant [48,49]. In this equation, *F*_0_, *F*, and *F*_∞_ are the fluorescence intensities in the absence of, at intermediate, and at full protein saturation, respectively; *K_d_* is the dissociation constant, *L* is the fixed ligand (DPH) concentration, and *P* is the added concentration of HSA. The association constant (*K_a_*) was calculated as 1/*K_d_*. The same experimental protocol was used to determine the binding constant with OG. In this case, the fixed amount of OG was 5.0 µmol L^−1^ and HSA 0–25.0 µmol L^−1^. The excitation was set at 305 nm, and the emission was 320 to 450 nm. The non-linear fitting was obtained using the GraphPad Prism version 8.00 for Windows (GraphPad Software, San Diego, CA, USA).

(2) $$\frac{F-F_{0}}{{F_{\infty }-F}_{0}}=\frac{\left[L\right]+[P]+K_{d}-\sqrt{{\left(\left[L\right]+[P]+K_{d}\right))}^{2}-4[L][P]}}{2[L]}$$

The interactions between HSA and quercetin or rutin were evaluated by quenching the intrinsic fluorescence of the protein. The excitation was set at 295 nm, and the emission at 310 to 410 nm. In these experiments, a fixed concentration of HSA (5.0 µmol L^−1^) was titrated with quercetin or rutin (0–5.0 µmol L^−1^). After each addition of the ligands, the mixtures were incubated for 2 min before the fluorescence measurements. The binding constants were determined by fitting the experimental data to Equation (3). In this equation, *F_0_* is the fluorescence in the absence of ligand; *F* is the fluorescence in the presence of ligand; *F_ratio_* (*F*/*F*_0_) is the observed fluorescence ratio; Φ is the fluorescence ratio change amplitude (1 − *F_ratio_*_∞_); *F_ratio∞_* is the ratio at an infinite concentration of the ligand; *P*_0_ is the fixed protein concentration; *L* is the concentration of added ligand (quercetin or rutin); *K_d_* is the dissociation constant; and *n* is the number of binding sites [50,51]. Since a 1:1 complex was assumed, *n* was set as 1. Therefore, *K_d_* and Φ were treated as the fitting parameters in the non-linear least-squares analysis. Before determining the binding constants, the fluorescent intensities were corrected for the inner filter effect (Equation (4)) caused by the attenuation of the excitation and emission signals provoked by the light absorption of the flavonoids. *F_corr_* and *F_obs_* are the corrected and observed fluorescence. *Ab_ex_* and *Ab_em_* are the absorptions of the mixture at excitation and emission wavelength, respectively. The absorbance spectra were measured using a Perkin Elmer Lambda 35 UV-visible spectrophotometer (Shelton, CT, USA), and the fluorescence spectra were obtained using a Perkin Elmer LS 55 spectrofluorometer (Shelton, CT, USA).

(3) $$F_{ratio}=1-\Phi [\frac{(K_{d}+nP_{0}+L)-\sqrt{{\left(K_{d}+nP_{0}+L\right)}^{2}-(4nP_{0}L)}}{2nP_{0}}]$$

(4) $$F_{corr}=F_{obs}\times 10^{\frac{\left({Ab}_{ex}+{Ab}_{em}\right)}{2}}$$

### 3.3. Circular Dichroism and Rayleigh Scattering Assays

The effect of 1,4-dioxane (20%) in the protein structure was evaluated by Far-UV-CD and Rayleigh scattering. The circular dichroism study was performed in a J-815 spectropolarimeter (Jasco, Japan) equipped with a thermostatically controlled cell holder and a 2 mm path-length quartz cuvette. The spectra were recorded after the incubation of HSA (2.0 µmol L^−1^) for 10 min in the presence or absence of 1,4-dioxane. The spectra were obtained with a scanning speed of 50 nm/min, a 1 nm step resolution, and a response time of 1 s. The baseline (aqueous solution with 20% 1,4-dioxane) was subtracted from all measurements. The elastic scattering of light, an indication of protein aggregation, was measured at 90° geometry on a Perkin Elmer LS 55 spectrofluorometer adjusted as follows: excitation at 350 nm and emission scanning from 300 to 400 nm. The reaction mixtures contained 5.0 µmol L^−1^ HSA in the presence or absence of 1,4-dioxane.

### 3.4. Time-Resolved Fluorescence Studies

The measurements were performed using a mini-tau filter-based fluorescence lifetime spectrometer coupled to a Time-Correlated Single Photon Counting (TCSPC) system (Edinburgh Instruments, Livingston, UK). The excitation was performed using a 370 ± 10 nm picosecond pulsed laser, and the emission was using a 375 ± 25 nm interference filter. The fluorescence decays were fitted using the multi-exponential decay model, Equation (5), and the average lifetime <τ> was calculated using Equation (6). The quality of the fit was assessed by χ^2^ values and residuals [52]. The excited-state lifetimes were determined using a fixed concentration of the DPH (5.0 µmol L^−1^) in the presence or absence of HSA (30 µmol L^−1^) in water or 20% 1,4-dioxane, 25 °C.

(5) $$I_{T}=\sum_{i=1}^{n}f_{i}e^{\frac{-T}{\tau_{i}}}$$

(6) $$<\tau >=\sum_{i=1}^{n}f_{i}\tau_{i}$$

### 3.5. Molecular Dynamics

The structure of human serum albumin (HSA) was obtained from the Protein Data Bank (PDB ID: 1AO6), while the structures of the small molecules were retrieved from PubChem: DPH (CID 5376733), OG (CID 61253), and 1,4-dioxane (CID 31275). To prepare the HSA structure, polar hydrogen atoms and Gasteiger charges were added using the AutoDockTools software [53]. Grids were generated using the AutoGrid program [53] with a spacing of 0.375 Å, centered visually on domain IIIB for 1,4-dioxane and on domain II for OG. The binding sites in HSA were investigated using AutoDock 4.2 and the Lamarckian Genetic Algorithm (LGA), with a population size of 150, while other parameters were set to default values. To explore different conformations, 100 docking runs were conducted. Final conformations were selected from the most negative energy structures within the most representative cluster [54] and visualized using the Visual Molecular Dynamics (VMD) software [55]. A diagram of interactions between the molecules and amino acids in the binding site was generated using LigPlot [56].

Molecular dynamics (MD) simulations of the albumin-DPH and albumin-OG complexes, both in 0% and 20% dioxane, were conducted with the GROMOS54a6 force field in Gromacs v.5.1.4 [57]. For simulations without dioxane, each complex was placed in a rectangular box, solvated with simple point charge (SPC) water, and neutralized with NaCl. In simulations with 20% dioxane, the number of dioxane molecules was calculated based on the box volume and inserted, followed by solvation with SPC water and neutralization with NaCl. Topologies for DPH, OG, and dioxane were generated using the ATB webserver [58].

Energy minimization was performed with the steepest descent method. Initial equilibration was conducted in the NVT ensemble for 100 ps, with temperature control provided by a V-rescale thermostat at 298 K and heavy atoms restrained with 1000 kJ mol^−1^ nm^−2^. The LINCS algorithm [59] was applied to constrain all bonds, a 1.4 nm cut-off was used for short-range non-bonded interactions, and long-range electrostatics were calculated using the particle mesh Ewald (PME) method [60]. A second equilibration step was performed in the NPT ensemble with a Parrinello–Rahman barostat for isotropic pressure control over 100 ps and the heavy atoms restrained. Finally, the restraints were removed and the systems were simulated for 40 ns. From these simulations, the fluctuations in RMSD and the distance between COM of binding site and the ligands were analyzed using *gmx rms* and *gmx distance*, respectively.

The final coordinate from previous step was used as starting point for pulling the ligand out of the binding site using steered molecular dynamics, with a spring constant of 1000 kJ mol^−1^ nm^−2^ and a pulling rate of 0.01 nm ns^−1^. The reaction coordinate was defined as the distance between DPH-Val408 and OG-Glu339. Sampling points were selected based on distance, with intervals of 0.1 nm up to 4 nm and 0.3 nm from 4 to 6 nm. These selected coordinates were subjected umbrella sampling [61], with 10 ns of molecular dynamics performed in each defined window. The potential of mean force (PMF) profile along the reaction coordinate was calculated using the weighted histogram analysis method (gmx wham) [62]. Statistical errors were estimated using bootstrap analysis, employing the method specified by the “b-his” flag. Each point along the reaction coordinate was represented by 1000 independent histograms, with random weights assigned to each.

## 4. Conclusions

The hydrophobic effect plays a critical role in organizing proteins and supramolecular systems, as well as in host/guest complexation. Through a new experimental approach, we demonstrated its significance in the interaction between hydrophobic ligands and HSA. By maintaining the protein and ligand fixed and altering the medium’s polarity, we have illustrated that the disparity in hydrophobicity between the protein’s microenvironment and the surrounding solvent is essential for the effectiveness of the interaction. Our experimental results were supported by molecular dynamics simulations, which indicated that the energy barrier for the ligands to lose contact with the hydrophobic site of the protein and transition into the surrounding solvent is lower when the medium is more hydrophobic. These results shed light on albumin–ligand interaction; usually, only the energetic factors involving the protein and the ligand, i.e., intermolecular forces, are considered in most studies. From our results, the entropic factor related to the remotion of the ligand of the bulk solvent to the protein cavity should always be considered. In conclusion, while the range of dielectric constants used in our study (ε = 80–63) extends beyond the exact values found in cellular environments, it provides a controlled framework to explore the influence of solvent hydrophobicity on ligand binding affinity. Although such extremes in polarity are unlikely to occur in bulk solution within cells, localized variations in hydrophobicity can exist, particularly near membrane interfaces or within protein complexes, where microenvironments might mimic the effects observed here. Thus, our findings suggest that even modest shifts in local hydrophobicity could modulate binding strengths in cellular contexts, offering insights into how environmental factors may regulate molecular interactions in vivo.

## Data Availability

The original contributions presented in the study are included in the article, further inquiries can be directed to the corresponding authors.
